# The UK Tobacco and Vapes Bill (2023/4): framing strategies used by tobacco and nicotine industry actors faced with an endgame policy (a generational sales ban of tobacco products) and nicotine product restrictions

**DOI:** 10.1136/tc-2024-059207

**Published:** 2025-03-23

**Authors:** Britta K Matthes, Tess Legg, Rosemary Hiscock, Allen W A Gallagher, Karin Silver, Hala Alaouie, Duncan Thomas, Anna B Gilmore

**Affiliations:** 1 Department for Health, University of Bath, Bath, UK

**Keywords:** Tobacco industry, Public policy, End game, Electronic nicotine delivery devices

## Abstract

**Background:**

In 2023, the UK government proposed a Bill introducing a generational ban on the sale of tobacco products and measures targeting youth nicotine product (NP) use. Industries’ responses remain unexplored.

**Methods:**

We analysed 43 consultation and evidence submissions from tobacco and nicotine industry-linked actors, assessing their connections to transnational tobacco companies (TTCs) and using an evidence-based taxonomy to examine framing strategies.

**Results:**

TTCs and actors with known current TTC links accounted for 42% of submissions. Other actors—some with historic TTC ties—were other tobacco products (OTPs) specialists, NP specialists and retailers. Tobacco and nicotine industries were generally portrayed as ‘good’ with TTCs framing themselves as public health champions, while policymakers, the public health community and non-compliant sellers were depicted as ‘bad’ actors. TTCs, linked groups and additional OTP actors opposed the generational ban, arguing it lacked evidence and would harm the economy and increase violence against retailers. Proposed alternative approaches included exempting heated tobacco products (HTPs) and OTPs, and raising the age of sale to 21. TTCs, linked actors and NP actors opposed restrictions on NP flavours, packaging and display, arguing these would infringe on rights, harm retailers, stifle innovation, and increase smoking and illicit trade. They framed youth NP use as resulting from irresponsible individuals and advocated for education, stricter penalties and self-regulation.

**Conclusion:**

When faced with tobacco endgame and nicotine control policies in the UK, tobacco and nicotine industries pushed back using framing strategies common to health-harming industries. Despite contradicting their ‘transformation’ narratives, TTCs still sought to position themselves as public health actors deserving policy access. Advocates, researchers and policymakers can anticipate opposition to endgame policies—such as a generational sales ban—and NP restrictions, proactively preparing to counter this opposition in order to safeguard public health policy from vested interests.

**WHAT IS ALREADY KNOWN ON THIS TOPIC**

- The tobacco industry has a long history of opposing public health policies using well-established arguments. Less is known about how its tactics have evolved post-‘transformation’ or how the nicotine industry engages in policymaking.
- The UK Tobacco and Vapes Bill, which proposes a generational ban on tobacco sales—unprecedented in implementation—and stricter nicotine product (NP) regulations provides a unique opportunity to address these research gaps.

**WHAT THIS STUDY ADDS**

- Consistent with established strategies used by health-harming industries, actors portrayed themselves as responsible while shifting blame for smoking and youth NP use onto individuals, reinforcing arguments for self-regulation, and individual- over population-based interventions.
- Core industry arguments remained predictable, such as claims that measures were unnecessary, economically harmful and would fuel illicit trade. Some were more policy-specific, such as claims that the generational ban would increase retailer-targeted violence and NP restrictions would drive cigarette smoking.

**HOW THIS STUDY MIGHT AFFECT RESEARCH, PRACTICE OR POLICY**

- Policymakers, advocates and researchers must recognise that despite deploying ‘transformation’ narratives and positioning themselves as legitimate public health and policy actors, transnational tobacco companies continue to oppose regulation.
- Given increasingly complex industry networks, stronger safeguards are needed to prevent vested interests from influencing public health policymaking.

## Introduction

In late 2023, the UK government announced plans to introduce an endgame policy (ie, a policy intended to phase out tobacco use) to create a smoke-free generation. Only two countries—Malaysia and New Zealand—have attempted to implement endgame policies, but neither has succeeded, with tobacco industry interference suspected to be a contributing factor.^1–3^ A very small but growing number of city administrations in the Philippines and the USA have adopted endgame policies, which include phasing out tobacco and nicotine product (NP) sales—often referred to as a generational sales ban (GSB) or smoke-free generational policy—outright bans or restrictions on the number of tobacco retailers.^1^ In Brookline, Massachusetts—the first town to adopt a GSB—legal action by tobacco companies was unsuccessful, encouraging other localities in the USA to consider similar policies.^4 5^ The UK government has also proposed a GSB, which would prohibit the sale of any tobacco products, including other tobacco products (OTPs) such as cigars and heated tobacco products (HTPs), to anyone born after 31 December 2008.

Youth use of NPs, particularly disposable electronic cigarettes (e-cigarettes, commonly known as vapes) has grown in recent years, including among those who have never smoked^6^ spurring the UK government to take action.^7^ The Tobacco and Vapes Bill^8^ (hereafter the Bill) introduced into the UK Parliament in March 2024,^9^ aimed to establish a GSB and to reduce NP appeal and availability to children. The government would have new powers to regulate NP flavours and contents, packaging and product requirements, and point of sale display. Separate national regulations banning disposable e-cigarettes are set to come into force in June 2025,^10^ and a tax on e-cigarette liquids was announced in October 2024, to take effect from October 2026.^11^

The Bill fell at Committee stage when a general election was called in May 2024. The new government reintroduced the Bill in early November 2024 with additional measures, including banning advertising of NPs and extending powers to ban smoking in certain outdoor areas.^12^

Investigations revealed that transnational tobacco companies (TTCs) had attempted to interfere with the policy process from the start in 2023, particularly around the GSB: they threatened the government with legal action and lobbied policymakers directly and via third parties.^1 13 14^ However, framing strategies—that is, the narratives and language actors use to shape public and policymakers’ perceptions and norms to align with their interests^15^—used to influence the Bill have not previously been systematically studied. This paper therefore aims to examine framing strategies employed by tobacco and nicotine industries and linked actors regarding the 2023/4 Bill.

Studying industry opposition to the Bill addresses three important knowledge gaps. First, although TTC political activity has been well documented,^16 17^ little research has examined whether this activity has changed since TTCs began increasingly marketing newer tobacco and NPs^18^ and voicing a commitment to ‘transformation’—defined as adopting public health goals and claiming to transition to selling ‘less harmful’ products.^19^ Recent research focuses on TTC discursive strategies on e-cigarette use^20 21^ and regulation^22 23^ but not on tobacco control. Second, interference in endgame policies has not been studied outside New Zealand.^24^ Finally, nicotine industry efforts to influence policy formulation have not previously been examined.

This work could support the passage of the 2024/2025 Tobacco and Vapes Bill; help countries pursuing similar legislation^25^ anticipate, recognise and counter industry arguments; and facilitate critical examinations of industry’s ‘transformation’ claims.

## Methods

### Data sources

To examine framing strategies of tobacco and nicotine industries, we drew on submissions made (1) to the Department of Health and Social Care (DHSC) public consultation held in late 2023^26^ and (2) to the House of Commons Public Bill Committee call for evidence in April 2024.^27^ The consultation contained 71 questions, including some on measures not included in the Bill (eg, disposable e-cigarettes, tax on NPs). It attracted almost 120,000 submissions of which around 90,000 were suspected bots.^28^

The call for evidence did not include specific questions and led to 56 written responses.^29^

### Data collection

Consultation responses were obtained from a journalist who had made a Freedom of Information request to DHSC for responses from 13 named organisations. Asking for a small number of responses was an attempt to avoid the request being denied for cost reasons. All 56 evidence submissions were downloaded from the UK Parliament website.

We excluded one consultation response and 25 evidence submissions from organisations without known links to tobacco or nicotine industries based on publicly available information identified through Google searches and websites including Tobacco Tactics (www.tobaccotactics.org). We did not consider declared industry links which were mandated only for consultation responses (but not for evidence submissions). 12 consultation responses and 31 evidence submissions were included.

### Data analysis

We categorised actor types in three ways: (1) by supply chain categories (eg, manufacturer, importer and distributor, retailer), and other types of organisations often associated with industry interference (eg, trade associations, think tanks);^30 31^ (2) by products manufactured or sold, namely cigarettes and other products (ie, TTCs), only OTPs, only NPs, or none; and (3) by known links to TTCs (current *direct* links, through funding, membership; current *indirect* links, via a third organisation; historic direct or indirect links; any other connections).

An evidence-based taxonomy of corporate political activity (CPA) detailing framing strategies commonly used by health-harming industries^30^ was used to guide our analysis. The taxonomy captures industries’ ‘good vs bad’ dichotomy to frame the actors, the problem and the solution(s) in ways that undermine the political acceptability of strong policies that threaten industry profits, reducing the likelihood of their adoption.^30^ To check the taxonomy’s relevance to our analysis and develop a common understanding of it, the coders (BM, TL, RH, AWG) conducted a pilot with five representative documents. To finalise the coding framework, two coders (BM, TL) double-coded eight other documents and, to ensure consistent application, all coders engaged in a live coding session. The remaining documents (35/43) were split between three coders (TL, RH, AWG) and the fourth (BM) double-coded at least three documents per coder (10/35). Each coder’s share included a mix of actor and document types. Coding was conducted in NVivo 14 (Lumivero). Coders regularly met to discuss, for example, potential additional codes.

### Ethics

This project received a favourable opinion from the University of Bath’s Biomedical Sciences Research Ethics Committee (Reference: 6196–7154).

### Patient and public involvement

No patient and public involvement.

## Findings

### Actors and their links to transnational tobacco companies (TTCs)

The 43 documents were submitted by 33 actors. One organisation sent three documents, and eight sent two each (see online supplemental file 1 for details). Some submissions from OTP actors contained similar or identical content.

The 33 actors were manufacturers, importers, retailers, trade associations, think tanks and under the ‘other’ category a smokers’ rights group, two organisations advocating for harm reduction, a consultancy and a retail magazine. Most were UK-based, except for two TTCs, one Danish OTP manufacturer, two Chinese NP manufacturers, two European trade associations and one US-based harm reduction advocacy organisation. Actors specialised in or represented the interests of a variety of products (table 1).

**Table 1:** Overview of actors and their current known TTC connections

Product specialisation actor type	Transnational tobacco companies (TTCs)	Actors only specialised in other tobacco products (OTPs)	Actors only specialised in nicotine products (NPs)	Other actors with TTC connections	*Number of actors*
Manufacturer	4	2 (1*)	2		***8***
Importer and distributor		3			***3***
Retailer		6		2†	***8***
Trade association	1‡	4 (1*)	1	1*	***7***
Think tank				2*	***2***
Other			3 (2*)	2*	***5***
***Number of actors***	***5***	***15***	***6***	***7***	***33***

* Actor with known current direct (ie, through funding or membership) or indirect (i.e., via a third organisation) links to TTCs (see online supplemental file 2 for details of TTC links).

† Actor with other current TTC connections (ie, commenting on the GSB in a TTC-sponsored press feature and at an event hosted by a TTC-funded actor) (see below for more information).

‡ Trade association with only TTC members (see online supplemental file 2).

GSB, generational sales ban.

Four actors were TTCs, and 10 had known current TTC links (one OTP manufacturer, three trade organisations, two think tanks, a smoker’s rights group, a retailer magazine and two harm reduction groups) (table 1). Six more actors had historic TTC links, including one OTP manufacturer, two OTP importers, two OTP trade associations and the NP trade association (see online supplemental file 2 for details of TTC links).

The two non-specialist retailers’ connections were through participation in a TTC-sponsored media article where both expressed reservations about the GSB.^32^ One also spoke at an event targeting MPs and journalists about the GSB’s impact on retailers.^33^ The event was organised by the TTC-linked smokers’ rights group, and other speakers included representatives from two TTC-linked organisations.^33^

The remaining actors had no links to TTCs identified.

### Framing strategies

In the following, we summarise actors’ framing strategies using the existing taxonomy. First, we outline how actors present themselves as ‘good’ (section 1) and portray others as ‘bad’ (section 2). Given the policy-specific nature of framing, we separately report strategies related to the GSB (section 3) and NP restrictions (section 4). For each of these measures, we outline (a) how the problem is framed, (b) why the proposed policy is considered ‘bad’ and (c) what ‘good’ alternatives are suggested. Detailed lists of framing strategies by actor types are provided in online supplemental file 3 (‘good’ and ‘bad’ actors), online supplemental file 4 (GSB) and online supplemental file 5 (NP restrictions).

#### We are the ‘good’ actors

TTCs, NP and OTP actors portrayed themselves as *legal entities* selling lawful products and complying with regulations. Manufacturers, OTP importers, OTP retailers and trade associations emphasised their role as *key economic actors*, highlighting their tax contributions and job creation. OTP actors also underscored that their products attract tourists who spend locally. Several OTP actors presented themselves as *part of the social fabric*, for example, as ‘family-run’ and ‘truly heritage British institutions’.

Several actors framed themselves as *legitimate scientific actors*, claiming to support evidence-based policies. TTCs, a retailer association and an NP manufacturer cited proprietary research, including expert reports and consultancy studies and the NP trade association referenced research of a member organisation.

To appear as *legitimate policy actors*, manufacturers variously acknowledged the health risks associated with smoking and concerns about youth NP use, and expressed a commitment to tackling the environmental impact of disposable e-cigarettes. In an apparent attempt to secure access to policymaking, one TTC offered to provide oral evidence, while a NP manufacturer expressed readiness to work with regulators. OTP actors emphasised retailers’ perspectives as ‘invaluable’ for policymaking.

Many framed themselves as *public health champions*: TTCs stated that they had developed products to ‘reduce harm,’ OTP retailers highlighted shifting away from cigarette sales ‘to benefit society’ and the NP trade association stressed the industry’s ‘public health benefits’. TTCs and NP manufacturers stated that they only target adults and work to tackle online youth access, non-compliant NPs and retailers; to educate regulators on nicotine pouches; and to recycle disposable e-cigarettes. Several actors also signalled support for the government’s ambition of protecting youth from NPs. Despite opposing the proposed measures, three TTCs and the NP trade association purported to support the government’s smoke-free 2030 ambition. One NP manufacturer supported the GSB.

Finally, NP and OTP actors presented *themselves as victims*, with the consultancy protesting its exclusion from providing oral evidence despite not being linked to the tobacco industry. OTP organisations criticised the government’s ‘aggressive […] interpretation’ of the WHO Framework Convention on Tobacco Control (WHO FCTC) as threatening their sector.

#### These are the ‘bad’ actors

Several actors *criticised policymakers’ skills and motives*: OTP actors described the government’s stance on the WHO FCTC as ‘inaccurate’, while a think tank argued that policymakers ‘still do not understand the concept of tobacco harm reduction’. There were allegations of an ‘unbalanced’ Bill Committee, an ‘undemocratic’ expert witness selection process and a ‘flawed’ consultation. Furthermore, a think tank accused DHSC staff of being ‘paternalist fantasists’ with ‘prohibitionist delusions’ and suggested—as did the smokers’ rights group—that the DHSC had already ‘made up its mind’.

A few actors—notably no manufacturers**—***questioned the expertise and motives of the public health community*. The NP trade association argued scientists providing oral evidence ‘present[ed] a very skewed message’. A think tank portrayed public health advocates as ‘mendacious fanatics of the anti-smoking lobby’ and criticised ‘e-cigarette prohibitionist’ Michael Bloomberg’s ‘anti-vaping advocacy’ claiming he funds ‘front groups’.

Finally, actors in most categories accused *other industry actors* of *irresponsible practices*, including selling unsafe counterfeit products. Several accused ‘rogue retailers’ and ‘low-quality shops’ of selling to minors.

#### The proposed generational sales ban of tobacco products

All TTCs (n=4) and actors with current TTC links (n=10), plus additional OTP specialists (n=13) and non-specialist retailers (n=2), argued against the GSB. NP actors without current TTC links supported the GSB or did not mention it (table 2).

**Table 2:** Framing strategies regarding the generational sales ban

Framing the policy space	Frame-supporting claims	Key illustrations (based on taxonomy in Ulucanlar et al., 2023, see online supplemental file 4 for details)
**The ‘trivial’ and ‘individual’ problem: created by a minority of actors**	Problems are not caused by Industry’s products/services	Products cause minimal problems: There is an insignificant health threat from OTP or HTP use Youth smoking is already low and falling OTPs are fundamentally different from cigarettes OTPs contribute to enjoyment of life
	Problems arise from consumption patterns of atypical minorities	Youth smoking linked to individual personality traits and negative life events
	Health harms are exaggerated	Problems with youth smoking are exaggerated since most people who smoke quit
**The unacceptable, ‘bad’ solution: whole population, statutory**	Policies are unnecessary & unacceptable	GSB and inclusion of HTPs and OTPs are unnecessary Existing regulation is sufficient and should be better enforced Policy is disproportionate to the problem Policy is out of line with other countries’ policies Policy amounts to sanctioned age discrimination Policy is not evidence-based
	Policies/policy formulation contravene norms, rules & laws	Government has not sufficiently consulted industry or other groups Government has failed to conduct comprehensive social and economic impact assessments. Policy is unconstitutional and impedes the rights of businesses and people who smoke Policy is illegal
	Policies will lead to losses for businesses, economy and society	Policy will be difficult or impractical to implement effectively Policy will reduce competitiveness and innovation Policy will lead to business closures and job losses Policy will negatively impact the wider economy Policy will negatively impact LMICs
	Policy will fail and have perverse consequences	Policy will not work: Prohibition of products will not and does not work Banning OTPs will not protect youth from smoking Policy will cause confusion and fear among consumers and retailers Policy will increase illicit trade *Other:* policy will criminalise retailers and adults buying for other adults *Other*: policy will push OTP sales online and thus make it more difficult to enforce age restrictions
**The acceptable, ‘good’ solution: individual-focused, corporate supported**	Solutions should target individuals, not whole populations	Solutions should help individuals to change their consumption behaviours—adults who smoke need to be given accurate information Targeted interventions to treat problematic consumption: Youth prevention education Stricter penalties for non-compliant retailers
	Solutions should be self-regulatory and not disrupt business	*Other*: Preferred solutions that cause less disruption to business: Exempt HTPs and OTPs Increase the age of sale to 21

GSB, Generational Sales Ban; HTP, Heated Tobacco Product; OTP, Other Tobacco Product.

##### GSB addressing a ‘trivial’ and ‘individual’ problem

Several actors argued that the *problem*—harm from tobacco—was *not caused by their products*. OTP actors argued that OTPs were ‘fundamentally different’ from cigarettes, for example, ‘enjoying a fine cigar […] is totally different from a hasty “fag” on the office steps’. HTPs were framed similarly, with one think tank calling them ‘safe and effective’.

A harm reduction group suggested that cigarette use among youth *results from patterns of an* *a-typical* *minority*, explaining that ‘[b]ad circumstances or emotional turbulence in teenagers’ lives often manifest in multiple risky behaviours, of which vaping […] is just one’.

The smokers’ rights group argued that the *problem was exaggerated* since it is a ‘myth’ that youth smoking ‘almost inevitably’ leads to life-long smoking.

##### GSB as an unacceptable, ‘bad’ solution

Several actors argued the proposed ban, including on HTPs and OTPs, was *unnecessary and unacceptable,* with one think tank calling it ‘arguably the most idiotic and illiberal policy any democratic government has devised in the twenty-first century’. In several cases, better enforcement of existing regulation was framed as preferable. An OTP importer stated that including OTPs ‘would be the equivalent of passing legislation to ban sales of vintage red wine in order to curb youth binge drinking’. A few actors also flagged that New Zealand reversed its endgame policy, according to a trade association ‘due to, amongst other, a flourishing black market’. An OTP manufacturer contended the GSB would ‘leav[e] the UK isolated in adopting this extreme and untested measure’. All TTCs argued that a ban would be discriminatory*,* with one characterising it as ‘sanctioned age-discrimination’. Several actors suggested that the proposed ban, particularly its inclusion of HTPs and OTPs, was not evidence-based.

Actors further portrayed the *ban and its formulation as contravening norms, rules and laws.* This included claims that the government had not sufficiently consulted interested parties. TTCs and OTP actors also argued that the impact assessment omitted a rationale for including HTPs and OTPs, and a TTC criticised the ‘incomplete and unsound modelling’. All TTCs, several connected organisations and additional OTP actors contended that the GSB would violate rights, with a trade association stating it would ‘overturn th[e] fundamental freedom (ie, right to smoke) for the first time in British history’. A think tank argued a similar ban was deemed unconstitutional in Indonesia, and TTCs argued that a GSB would violate the European Union's Tobacco Products Directive and international trade agreements.

Many actors underscored ways in which the ban would *lead to losses for businesses, economy and society.* They referenced difficulties in implementing the ban (including preventing proxy sales) for retailers, police and trading standards and argued it would cause business closures and job losses. OTP actors argued that the GSB would ‘destroy (the OTP) market’ and negatively affect the UK’s reputation, setting it back ‘on the world stage’. Several actors referred to negative effects for tax revenue and tourism. An OTP actor argued that a ban would harm Central American cigar producers.

All TTCs, and many linked actors, maintained the GSB would *fail and have perverse consequences*. Many argued that the prohibition of products does not work or would not have worked elsewhere, with Bhutan and South Africa cited as jurisdictions where tobacco bans have supposedly increased illicit trade. All TTCs argued that a GSB on all tobacco products would stop adults who smoke from switching to ‘less risky’ HTPs by compounding misunderstandings about the relative risks of cigarettes and newer tobacco and nicotine products. Several actors, including TTCs, linked groups and a retailer stated that the ban would lead to fear among retailers, with a trade association speaking of an ‘escalation in more threatening anti-social behaviour towards shopkeepers and their staff, which is already at epidemic levels’. Several actors argued a GSB would increase illicit trade. Some mentioned the inadvertent criminalisation of retailers and adults buying tobacco products for other adults and argued that OTP sales would be pushed online, complicating enforcement of age restrictions.

##### The acceptable, ‘good’ alternative solutions to a GSB

Proposed alternative interventions *targeted individuals rather than whole populations*, including education campaigns to support switching to ‘less harmful’ alternatives, youth prevention education and stricter penalties for non-compliant sellers.

Additionally, actors argued for solutions that would cause *less disruption to their business.* All TTCs and several linked actors argued that HTPs should be exempt from the ban, with some referring to New Zealand as an example. Almost all OTP-linked actors suggested the same for OTPs. TTC-linked actors also asked to legalise snus which one stated ‘has been instrumental in Sweden’s remarkable journey towards becoming virtually smoke-free’. Additionally, several actors—including three TTCs—advocated for an increase in the tobacco age of sale to 21.

### Proposed measures to tackle nicotine product use among youth

All TTCs (n=4), several actors with current TTC links (n=6) and all other NP-specialised actors (n=4) argued against banning flavours, standardised packaging and/or display restrictions for NPs (table 3). The tobacco manufacturers’ trade association, all OTP-linked actors and the two non-specialist retailers did not comment on NP-related measures.

**Table 3:** Framing strategies regarding nicotine product restrictions

Framing the policy space	Frame-supporting claims	Key illustrations (based on taxonomy in Ulucanlar et al., 2023, see online supplemental file 5 for details)
**The ‘trivial’ and ‘individual’ problem: created by a minority of actors**	Problems are not caused by Industry’s products/services	Products cause minimal problems: NPs are safer than cigarettes and are important for smoking cessation Our products have been confused with other, genuinely harmful ones Illegal NPs and those selling NPs to children are the problem
	Problems arise from consumption patterns of atypical minorities	Youth smoking linked to individual personality traits and negative life events
	Health harms are exaggerated	n/a
**The unacceptable, ‘bad’ solution: whole population, statutory**	Policies are unnecessary and unacceptable	Existing regulation is sufficient and should be better enforced Policy disproportionate to the problem Policy not evidence-based
	Policies/policy formulation contravene norms, rules and laws	Industry and others were not sufficiently consulted Policy impedes the rights of businesses and individuals who smoke Policy is illegal
	Policies will lead to losses for businesses, economy and society	Policy will be impossible to implement (cost)effectively Policy will reduce competitiveness, innovation and investment and lead to business closures and job losses Policy will have negative impacts on business and will affect the wider economy
	Policy will fail and have perverse consequences	Policy will not work or has not worked elsewhere. Policy will lead to an increase in smoking Policy is a blunt or simplistic instrument and will not achieve nuanced change Policy will cause confusion or fear among adults who smoke Policy will increase illicit trade
**The acceptable, ‘good’ solution: individual-focused, corporate supported**	Solutions should target individuals, not whole populations	The solution is to help individuals or ‘problem’ sub-populations Adults who smoke need to be given accurate information The solution is to ‘treat’ problematic consumption using targeted interventions and ‘harm reduction’ approaches Youth should be educated about harm reduction Communities should be educated about illegal e-cigarettes Non-compliant retailers should be penalised (more) severely
	Solutions should be self-regulatory and not disrupt business	Self-regulation and voluntary action are more effective Flavours should be a matter of market forces We should build on existing self-regulatory frameworks

NP, Nicotine Product .

#### NP restrictions addressing a ‘trivial’ and ‘individual’ problem

TTCs and NP manufacturers and linked actors argued that the *problem*—youth NP use—was *not caused by their products,* instead emphasising that e-cigarettes and nicotine pouches are ‘safer’ alternatives to cigarettes and important in smoking cessation. Rather than their products, illegal products and underage sales cause harm.

A harm reduction group associated e-cigarettes with consumption *patterns of an* *a-typical* *minority*. Similarly, the consultancy stated that most youth NP use stems from curiosity, not addiction.

#### NP restrictions as an unacceptable, ‘bad’ solution

Several actors contended that measures such as flavour bans, standardised packaging, and display restrictions were *unnecessary and unacceptable*. Most TTCs, linked actors and all NP actors advocated instead for stricter enforcement of age-of-sale regulations. Some claimed that NPs should not be regulated like tobacco products, as they were ‘less harmful’. One harm reduction group stated that ‘despite the significant public health harms of alcohol, there are no widespread calls for restrictions on alcoholic drink flavours’. To underscore their concerns, several actors argued that the proposed measures were not evidence-based.

Actors also claimed that the *policy and process contravened norms, rules and laws*. This included concerns about insufficient consultation. Additionally, a TTC and TTC-linked actors expressed that the measures would infringe on businesses’ and individuals’ rights and freedoms, pointing, for example, to companies’ rights to distinguish their products and ‘adult smokers’ right of choice’. Another TTC argued that flavour, packaging and display restrictions would violate intellectual property rights and international trade agreements.

It was further argued that NP restrictions would lead to *losses for businesses, economy and society*. Several actors—but no retailers—claimed a display ban would be costly for retailers. It was also argued that a display ban would lead the nicotine industry to become dominated by tobacco companies and disproportionately affect small and new businesses. TTCs and linked actors warned that removal of branding and display would discourage product improvement and make specialist shops ‘unviable’, leading to job losses.

Finally, TTCs, linked organisations and all NP actors argued that the *measures would fail and have perverse consequences*, even leading to increased cigarette smoking; flavour bans had not succeeded elsewhere; and standardised packaging and display restrictions would confuse (potential) users, especially if NPs were regulated like tobacco products. TTCs and linked actors stated that flavour and packaging restrictions would increase illicit trade.

#### The acceptable, ‘good’ alternative solutions to NP restrictions

TTCs, NP manufacturers and linked actors proposed alternative approaches to NP restrictions, *targeting individuals rather than whole populations.* A harm reduction organisation proposed point-of-sale educational material. One TTC suggested NP packaging regulation to include ‘Government-approved messaging on the risk profile of [e-cigarettes] compared with combustible cigarettes and the benefits of transitioning’. To address youth NP use, actors suggested educating pupils about harm reduction, raising community awareness about illegal NPs and sales and stricter penalties for non-compliant NP sellers.

Actors also argued for *self-regulation and approaches not disrupting business.* A harm reduction organisation argued that self-regulatory frameworks could effectively address youth appeal. For nicotine pouches, a TTC urged the adoption of a voluntary industry framework into legislation. The NP trade association demanded statutory consultations for any further measures.

## Discussion

This is the first study examining industries’ responses to the UK Tobacco and Vapes Bill (2023/4). It shows that tobacco and nicotine companies and linked actors used framing strategies common across health-harming industries.^30^ Despite opposing GSB and/or NP restrictions, they presented themselves as the ‘good’ actors, denying responsibility for smoking or youth NP use, while questioning policymakers’ and public health actors’ skills and motives and blaming individuals’ irresponsible behaviours. The proposed solutions—GSB and NP restrictions—were opposed, using familiar industry claims: that both were unnecessary, violated trade agreements, harmed the economy and fuelled illicit trade. Other, more measure-specific arguments were raised, including claims that the GSB would increase violence against retailers and that NP restrictions would raise smoking rates.

Proposed alternatives aligned with industries’ interests. Raising the age of sale to 21 and excluding HTPs and OTPs from the GSB would protect tobacco companies, including TTCs and OTP specialists. Raising the age of sale would avoid ending tobacco sales completely; exemptions would allow continued HTPs and OTPs sales which companies could seek to increase in the light of the GSB. Education- and penalisation-focused approaches to address smoking and youth NP use would cause less business disruption while possibly opening the door to less effective, industry-led solutions. For example, the tobacco industry has long promoted its youth smoking prevention programmes—which have failed to reduce youth smoking—to avoid regulation.^34^ This tactic is mirrored by other health-harming industries, including alcohol^35^ and gambling.^36^

This study reveals how the four TTCs and their linked actors—10 with known current and six with known historic ties—strategically deployed arguments to position TTCs as legitimate policy and public health actors. For example, OTP actors emphasised the GSB’s impact on their sector, while TTCs, consistent with previous studies,^16 17^ avoided acknowledging negative consequences for their business. Additionally, third parties, rather than TTCs themselves, were the primary actors criticising and attacking policymakers and the public health community.

Unlike during standardised packaging debates^37 38^—the last major UK policy change—TTCs have not launched an extensive media campaign. This may be because doing so would openly contradict their ‘transformation’ narratives or due to the widespread public, political and expert support for the GSB.^39–41^ It is also likely linked to the nature of the endgame policy: in New Zealand, where the proposed policy aimed, among other measures, to drastically reduce the number of tobacco retailers, a TTC-funded campaign sought to ‘save’ retailers.^24^

This study has limitations: available consultation data lacked submissions from OTP actors and non-specialist or NP retailers. Full disclosure of responses—which should happen in a timely manner^42^—would enable further analyses. The 90,000 suspected bot submissions also warrant scrutiny, as research on bots in policymaking has primarily examined government use.^43^

This study underscores the growing complexity of tobacco and NP regulation and industry networks which obscure connections. For example, an OTP trade association with TTC membership^44^ speciously stated that its members were ‘mainly smaller and mid-sized tobacco companies…[and] represent a tobacco entrepreneurship whose values are tied to traditional products and a longstanding cultural heritage’. To enhance transparency in line with the implementation guidelines for Article 5.3 of the WHO FCTC,^45^ written evidence submissions should, as a first step, require full disclosure of tobacco industry links, along with consideration of the accuracy of these disclosures or the absence thereof. Of the five organisations currently linked to TTCs whose consultation responses we had access to, one—a harm reduction advocacy group—denied any direct or indirect tobacco industry ties, despite being founded and funded by a lobby organisation that accepted TTC funding and having received direct TTC funding itself.^46 47^ The same group also stated in the EU transparency register to ‘not represent commercial interests’.^48^

UK advocates and researchers must continue monitoring opposition actors and arguments, including attempts to redefine ‘smoke-free’ to align with TTCs’ ‘transformation’ claims, and alert policymakers to efforts aimed at weakening or undermining the November 2024 Bill. Since the arguments against GSB and NP restrictions largely mirror those used against previous measures, existing resources and evidence can help anticipate and counter opposition.^49^ At the same time, examining industry conduct in the context of quieter public opposition requires more investigative approaches, which could be supported by collaborations with journalists. Policymakers need to be aware of industry narratives and resist calls for exemptions for HTPs, OTPs or nicotine pouches, as such carve-outs create regulatory loopholes (eg, post-menthol cigarette ban marketing of menthol cigarillos).^50^

Though contexts differ, policymakers, advocates and researchers in other jurisdictions may find this study useful in preparing for likely tobacco and nicotine industries’ strategies, particularly when pursuing a GSB or stringent NP regulation.

## Conclusion

This study highlights how tobacco and nicotine industries strategically frame opposition to resist tobacco endgame and nicotine control measures, such as the UK Tobacco and Vapes Bill. Since the counterarguments largely echo those raised against previous policies, advocates and researchers can debunk the TTCs’ ‘pseudo-transformation’^19^ and assist policymakers in anticipating and preparing for likely challenges. This proactive approach can help ensure that endgame policies—including GSBs—and NP regulations face less resistance, facilitating smoother adoption and implementation.

## Supplementary material

10.1136/tc-2024-059207 online supplemental file 1

10.1136/tc-2024-059207 online supplemental file 2

10.1136/tc-2024-059207 online supplemental file 3

10.1136/tc-2024-059207 online supplemental file 4

10.1136/tc-2024-059207 online supplemental file 5

## Data Availability

Data are available upon reasonable request.
