# Supplementary material for: The UK Tobacco and Vapes Bill (2023/4): framing strategies used by tobacco and nicotine industry actors faced with an endgame policy (a generational sales ban of tobacco products) and nicotine product restrictions
Source: Tob Control. 2025 Mar 23;35(4):e059207. doi: 10.1136/tc-2024-059207 (PMC13479673; doi:10.1136/tc-2024-059207)
Supplement: Supplementary file 1 [file tc-35-4-s001.pdf]

Supplemental file 1: Actors by submission type

Table 1: Submissions by actor types

	Number of actors in the dataset responding to…		
	…public consultation	… call for evidence (committee stage)	…public consultation and/or call for evidence
**Manufacturers**			**8**
- Transnational tobacco companies (TTCs)	4	4	4
- Other tobacco companies (selling other tobacco products (OTPs))		2	2
- Nicotine industry	2		2
**Importers and distributors**			**3**
- OTPs		3	3
**Retailers**			**8**
- OTP specialist		6	6
- General		2	2
**Trade associations**			**7**
- Tobacco manufacturers		1	1
- Nicotine industry	1	1	1
- OTP manufacturers, importers, and distributors		3	3
- Retailers		1	1
- OTP retailers		1	1
**Other actors with known current TTC**			**6**
**links**	2		2
- Think tanks	1		1
- Smokers' rights group	2		2
- Organisations advocating for harm reduction		1	**1**
- Retail magazine			
**Other**			**1**
- Consultancy working in nicotine industry		1	1
**Total**	12	26	33
