# Supplementary material for: The UK Tobacco and Vapes Bill (2023/4): framing strategies used by tobacco and nicotine industry actors faced with an endgame policy (a generational sales ban of tobacco products) and nicotine product restrictions
Source: Tob Control. 2025 Mar 23;35(4):e059207. doi: 10.1136/tc-2024-059207 (PMC13479673; doi:10.1136/tc-2024-059207)
Supplement: Supplementary file 2 [file tc-35-4-s002.pdf]

Supplemental file 2: Connections between TTCs and actors

Table 1: Actors with links to TTCs

Actor type	Known current or historic TTC link?	Nature of TTC link	Source
**OTP manufacturer**	Current	Joint membership in a trade association	(1)
**OTP manufacturer**	Historic	Joint membership in a trade association	(2)
**OTP importer and distributor**	Historic	Joint membership in a trade association	(2)
**OTP importer and distributor**	Historic	Joint membership in a trade association	(2)
**Trade association representing tobacco manufacturers**	Current	Exclusive TTC membership	(3)
**Trade association representing OTP manufacturers**	Current	TTC members	(1)
**Trade association representing OTP manufacturers**	Historic	TTC members	(4)
**Trade association representing OTP distributors**	Historic	TTC members	(2)
**Trade association representing nicotine industry**	Historic	TTC members	(5)
**Trade association representing retailers**	Current	TTC members	(6)
**Think tank**	Current	Long-term TTC connection	(7)
**Think tank**	Current	Long-term TTC connection	(8)
**Smokers' rights group**	Current	Long-term TTC connection	(9)
**Retail magazine**	Current	TTC sponsors an annual award	(10)
**Group advocating for harm reduction**	Current	Received funding from TTC-linked third parties and received TTC funding	(11, 12)
**Group advocating for harm reduction**	Current	Received funding from TTC-linked third parties	(13)

References:

1. ESTA. Members. 2024. Available from: https://web.archive.org/web/20240713222741/https://www.esta.be/members/, accessed on 15/11/2024.
2. ITPAC. The Scottish Government's Consultation ofn Electronic Cigarettes and Strenghenting Tobacco Contol in Scotland - consulation responses from Imported Tobacco Products Advisory Council (ITPAC). 2015. Available from: https://web.archive.org/web/20241119225155/https://www.gov.scot/binaries/content/documents/govscot/publications/consultation-analysis/2015/05/consultation-electronic-cigarettes-strengthening-tobacco-control-responses/documents/0142-imported-tobacco-products-advisory-council-itpac/0142-imported-tobacco-products-advisory-council-itpac/govscot%3Adocument/00476419.pdf?inline-true. accessed on 19/11/2024.

3. TMA. Tobacco Manufacturers' Association. 2024. Available from: https://web.archive.org/web/20241120125906/https://www.the-tma.org.uk/, accessed on 20/11/2024.
4. TobaccoTactics. European Cigar Manufacturers Association. 2021. Available from: https://www.tobaccotactics.org/article/european-cigar-manufacturers-association/, accessed on 12/11/2024.
5. TobaccoTactics. UK Vaping Industry Association (UKVIA). 2024. Available from: https://www.tobaccotactics.org/article/uk-vaping-industry-association-ukvia/, accessed on 10/11/2024.
6. ACS. Premier Club. 2024. Available from: https://web.archive.org/web/20241001094210/https://www.acs.org.uk/about-us/suppliers/premier-club, accessed on 10/11/2024.
7. TobaccoTactics. Institute of Economic Affairs. 2024. Available from: https://www.tobaccotactics.org/article/institute-of-economic-affairs/, accessed on 10/11/2024.
8. TobaccoTactics. Adam Smith Institute. 2024. Available from: https://www.tobaccotactics.org/article/adam-smith-institute/, accessed on 10/11/2024.
9. TobaccoTactics. Forest. 2020. Available from: https://www.tobaccotactics.org/article/forest/, accessed on 10/11/2024.
10. Asian Trader Awards. Our sponsors. 2024. Available from: https://web.archive.org/web/20241115195628/https://demo.asiantraderawards.co.uk/sponsorship/ accessed on 15/11/2024.
11. TobaccoTactics. World Vapers' Alliance. 2024. Available from: https://www.tobaccotactics.org/article/world-vapers-alliance/, accessed on 10/11/2024.
12. Bredderman W, Sollenberger R. Guess Who's Secretly Backing This 'Anti-Smoking' Vape Group2022. Available from: https://www.thedailybeast.com/world-vapers-alliance-slams-cigarettes-big-british-american-tobacco-is-secretly-behind-it/, accessed on 05/02/2025.
13. TobaccoTactics. Knowledge-Action-Change. 2023. Available from: https://www.tobaccotactics.org/article/knowledge-action-change/, accessed on 10/11/2024.
