# Supplementary material for: The UK Tobacco and Vapes Bill (2023/4): framing strategies used by tobacco and nicotine industry actors faced with an endgame policy (a generational sales ban of tobacco products) and nicotine product restrictions
Source: Tob Control. 2025 Mar 23;35(4):e059207. doi: 10.1136/tc-2024-059207 (PMC13479673; doi:10.1136/tc-2024-059207)
Supplement: Supplementary file 3 [file tc-35-4-s003.pdf]

Supplemental File 3: The ‘good’ and ‘bad’ actors

Table 1: Corporations as the ‘good’ actors: Frame-supporting claims and illustrations (based on Ulucanlar et al., 2024)

Frame-supporting claims	Illustrations	Types of actors using claims
**We are legal entities**	<u>Corporations have a right to conduct their business and to trade and abide by laws and regulations</u>	
	The tobacco industry, is a regulated, taxed and **legal industry** that sells lawful products	TTC and trade association (tobacco manufacturer)
	OTP companies sell legal products and have always adapted to comply with the law – “we are Good Citizens!”	OTP importer and retailers
**We are a key economic actor**	<u>Corporations are engines of economic growth and future prosperity</u>	
	**Taxpayers**	
	- Tobacco companies are key taxpayers.	TTC, trade association (tobacco manufacturer)
	- OTP businesses contribute taxes	OTP manufacturer, trade association (importer), OTP retailers
	**Job creators**	
	- Tobacco companies provide jobs	TTC
	- OTP companies “employ hundreds of staff” directly and indirectly	OTP manufacturer, OTP retailers, trade association (OTP manufacturers)
	- OTP sector main source of employment in rural areas	Trade association (OTP manufacturer)
	- Nicotine industry generating jobs across the wider economy	NP manufacturer
	OTPs attract **tourists**	OTP importer, OTP retailers, trade association (OTP importers)
	OTP actors are key part of local economy	OTP retailer
**We are part of the social fabric**	<u>Corporations are socially embedded in country/region and part of its history.</u>	
	OTP sector is part of **local heritage history**, deeply rooted in local economies, communities and national heritage”.	OTP manufacturer, trade associations (OTP manufacturers)
	OTP businesses **are family-run**	OTP manufacturer, OTP importers, OTP retailers, trade associations (OTP manufacturers, importers)
	OTPs are **cultural products with traditional heritage**, “envy of the world” and OTPs retailers “truly heritage British institutions”	OTP trade association, retailers
**We are a legitimate policy actor**	<u>We understand need to tackle issue, are reasonable and are willing to enter into partnership with government.</u>	
	- We acknowledge the health risks associated with smoking and concerns with youth vaping	TTC, OTP manufacturer, NP manufacturer
	- We have a collective goal with policymakers	
	- We are happy to provide evidence in Committee and work with regulators.	OTP trade association<br>TTC, NP manufacturer
	<u>We have expertise and information that government needs in making policy</u>	

	- OTPs retailers have invaluable expertise and experience given that they will be directly impacted by the proposed policy	OTP retailers, trade association (OTP)
	<u>We need access to policy spaces and decisionmakers because we are part of the solution</u>	
	- The responsibility for tackling environmental impact of disposable e-cigarettes lies with **all stakeholders**	NP manufacturer
**We are a legitimate scientific actor**	<u>Corporations support evidence-based policy</u>	TTCs, OTP and NP manufacturers, OTP importer, trade associations (tobacco manufacturers, NP), organisation advocating for harm reduction, NP consultancy
	<u>Corporations have expertise in the science of product health harms and solutions.</u>	
	- We conduct our own research/ commission experts/ publish peer-reviewed papers	TTCs, NP manufacturer, trade associations (retailers, NP)
	<u>Corporations are valuable educational resources to the public health community</u>	n/a
**We are a champion of public health**	<u>Corporations are responsible, committed to prevention of NCDs and working to reduce health harms</u>	
	- To reduce harm, we are developing alternative products	TTCs
	- We have moved away from selling cigarettes "to benefit society"	OTP retailers
	- The nicotine industry benefits public health	Trade association (NP)
	- As a responsible manufacturer, we are only targeting adult consumers	TTCs and NP manufacturers
	- We take additional measures to ensure our products are only sold to adults online	TTC
	- We are educating regulators about nicotine pouches	TTC
	- We are working on clamping down on illegal e-cigarettes and bad actors, engaging in campaigns	NP manufacturer, trade association (NP)
	<u>Corporations support the proposed policy</u>	
	- We support government's smoke-free 2030 ambition	TTCs, trade association (NP)
	- We support the generational ban	NP manufacturer
	- We support government restrictions protecting youth from accessing NPs	TTCs, NP manufacturer, trade associations (retailers, NP)
**We are socially responsible**	<u>Corporations are committed and essential to sustainable development</u>	
	We, the NP industry, are engaging in initiatives on recycling of disposable vapes to minimise their impact on the environment	NP manufacturers and trade association
	<u>Corporations create welfare by investing in social and economic development and are concerned with social justice.</u>	n/a
**We are the victim**	<u>Corporations are unfairly demonised.</u>	
	- We, OTP actors, are different from Big Tobacco as our products don't encourage youth uptake and the government interpretation of the WHO FCTC is "aggressive"	OTP importer, OTP retailers, trade associations (OTP importers, OTP retailers)
	- Despite not having no links to the tobacco industry, we were still not invited to provide oral evidence to the committee	NP consultancy

Table 2: The 'bad' actors: Frame-supporting claims and illustrations

Frame-supporting claims	Illustrations/Examples	Types of actors using claims
**Policymakers who support unfavourable policies have questionable skills and motives**	Policymakers may have good intentions but are incompetent/misguided, offering policies that contradict existing policies, are ineffective, illegal or not in keeping with international norms and standards.	
	- Committee only consists of MPs that supported the Bill in 2nd reading and selection of those giving oral evidence was unbalanced	OTP retailers, trade associations (tobacco manufacturers, OTP importers, OTP retailers, NP),
	- The WHO FCTC was interpreted inaccurately	OTP importer, OTP retailers, trade associations (OTP importers, OTP retailers)
	- The government claims to be committed to evidence-based policymaking but failed to adequately consider evidence on OTPs	OTP importers, OTP retailers and trade association (OTP)
	- Government does not understand concept of harm reduction	Think tank
	- Government consultation process flawed, e.g., making assumption, not giving enough space to express views.	TTC, think tanks, organisations advocating for harm reduction.
	Policymakers are disingenuous, for example, they want to raise revenue, not protect the public's health, or have a hidden agenda, for example to introduce restrictions on other products or industries	
	- Staff from the Department for Health are "paternalistic fantasists" and "insane" and have "prohibitionist delusions". Policy is an "ideological crusade" and "ludicrous" and the government has already made up its mind prior to the consultation	Think tank, smokers' rights group
	- Delegation of power undermines parliamentary scrutiny of action	TTC
	- NP regulation is a slippery slope	Think tank
	Policymakers are authoritarian and want to control people's lives	
	- Policy "grotesque" and "abuse of power"	Smokers' rights group
**Public health community have questionable skills and motives**	Scientists are incompetent or untrustworthy, engaging in bad scientific practices and promoting false or misleading findings	
	- Oral evidence was "either misleading, incomplete, unsubstantiated, or just incorrect", sending a "skewed message"	Trade association (NP)
	Scientists and advocates are ideologically motivated and have anti-industry/anti-free-enterprise agenda	
	- The "anti-smoking lobby"/ "public health "industry" consists of "mendacious fanatics"	Think tank
	Scientists and advocates are fanatical and want to control the lives of the reasonable/responsible majority	
	- E-cigarette "prohibitionist" Michael Bloomberg "pumped hundreds of billions of dollars into anti-vaping advocacy worldwide", setting up "Bloomberg front groups"	Think tank

Other industry actors are irresponsible and don't comply*	*Other actors are engaging in questionable practices.*<br>- There are irresponsible manufactures, who engage in irresponsible marketing practices and produce counterfeit versions of products<br>- Irresponsible retailers selling to children facilitate youth access<br>- There are organised criminal gangs importing illegal products, e.g., "flooding the UK market with illegal vapes"	TTCs, NP manufacturers, trade association (NP), NP consultancy<br>TTCs, NP manufacturers, trade association (NP, retailers), retailers<br>NP manufacturers, retailer, retail magazine

*This frame-supporting claim was not part of the original framework but was added inductively during data analysis.
