# Supplementary material for: The UK Tobacco and Vapes Bill (2023/4): framing strategies used by tobacco and nicotine industry actors faced with an endgame policy (a generational sales ban of tobacco products) and nicotine product restrictions
Source: Tob Control. 2025 Mar 23;35(4):e059207. doi: 10.1136/tc-2024-059207 (PMC13479673; doi:10.1136/tc-2024-059207)
Supplement: Supplementary file 4 [file tc-35-4-s004.pdf]

Supplemental File 4: Actors' framing strategies related to a generational sales ban

Table 1: Framing strategies regarding a generational sales ban: the problem, the 'bad' solution and the 'good' solution(s) (based on Ulucanlar et al., 2024)

Framing the Policy Space	Frame-supporting claims	Illustrations/Examples	Types of actors using claims
**The 'trivial' and 'individual' problem: created by a minority of consumers and other actors**	Problem is not caused by industry's products/services	Products/ingredients/services are harmless or cause minimal problems	
		- There is no significant health threat from OTP or HTP use	TTCs, OTP importer, OTP retailers, trade association (OTP manufacturer)
		- OTPs do not attract youth	OTP importer, OTP retailers, trade associations (OTP manufacturer, OTP)
		- Youth smoking is already low and falling	TTC
		Industry's products/ingredients have been misclassified / confused with other, genuinely harmful products/ingredients	
		- Cigars and other OTPs are a lifestyle product and are fundamentally different from cigarettes	OTP importers, OTP retailers, trade associations (OTP manufacturers, OTP importers)
		Industry's products/services contribute to health, wellbeing and enjoyment of life	
		- People consumer OTPs for pleasure and have no desire to quit	OTP importers, OTP retailers, trade associations (OTP manufacturers, OTP importers), smokers' rights group
		Health problems have complex causes that cannot be traced to industry products or services alone.	n/a
		Industry's products/services are aligned with cultural norms and practices and are used responsibly by the majority	n/a
	Problem arises from consumption patterns of atypical minorities	Health harms result from cognitive problems or physical/mental health problems	
		- Youth uptake of combustible cigarettes and newer products linked to "personality traits" and "emotional turbulence" in teenagers' lives	Organisation advocating for harm reduction
		Health harms result from individuals' wrong or uninformed choices and irresponsible behaviours	n/a
	Health harms are exaggerated	Problems including health harms are exaggerated	
		- Youth smoking does not inevitably lead to life-long smoking	Smokers' rights group
		There are far more serious and urgent health problems that government should prioritise instead	n/a

**The unacceptable, 'bad' solution: whole population, statutory**	Policies are unnecessary and unacceptable	Policy is unnecessary<br>- A sales ban is unnecessary and illogical, particularly in relation to HTPs and OTPs	TTCs, OTP manufacturers, trade association (OTP manufactures), think tanks, organisation advocating for harm reduction, retail magazine
		Existing regulation is sufficient and should be better enforced before new measures are introduced:<br>- Current measures are sufficient to reduce youth smoking and allow adults to make informed choices about consuming OTPs	OTP manufacturer, trade association (OTP), organisation advocating for harm reduction
		Policy is disproportionate to the problem<br>- A sales ban is an extreme policy measure	TTCs, OTP manufacturer, trade association (OTP)
		Policy is out of line with global standards and other countries' policies<br>- The UK is alone in adopting the measure, New Zealand recently reversed the decision	TTC and OTP manufacturer
		Policy is regressive and discriminatory<br>- Policy amounts to discrimination of adults born after a certain date and "infantilises" them	TTCs, OTP manufacturer, trade association (OTP importer) think tank, smokers' rights group
		Policy is not evidence-based<br>- Lack of evidence for including HTPs and OTPs in a sales ban	TTCs, OTP manufacturer, OTP importer, trade association (OTP), think tank, organisation advocating for harm reduction
	Policies / policy formulation contravene norms, rules and laws	Government has not sufficiently consulted industry or other groups	OTP importer, trade association (OTP), retail magazine (see also supplemental file 2)
		Government has failed to conduct comprehensive social and economic impact assessment<br>- Government impact assessment failed to consider impact on OTP importers/retailers and incomplete modelling on HTPs	TTCs, OTP importer, OTP retailers, trade association (OTP importers, OTP)
		Policy is unconstitutional, impedes basic rights incl. the right to free speech and curtails basic freedoms of a legal business<br>- Takes away the right to use tobacco products for adults who smoke and impedes corporate rights	TTCs, OTP manufacturer, OTP importer, OTP retailer, trade association (tobacco manufacturers, OTP manufacturers, OTP), think tank, smokers' rights group
		- Indonesia ruled it unconstitutional	Think tank
		Policy is illegal (e.g. it violates terms of international trade and investment agreements)<br>- Sales ban violates Tobacco Products Directive (TPD) and International Trade Agreements	TTCs
		The body proposing regulation has no legal authority to do so	n/a

	Policies will lead to losses for businesses, economy and society	Policy will be *difficult or impractical* to implement effectively<br>- Enforcement (including prevention of proxy sales) will be difficult/impractical/expensive for retailers, police and Trading Standards<br>-	TTCs, OTP retailer, retailer, trade associations (tobacco manufacturers, retailer) think tank, smokers' rights group
		Policy will reduce competitiveness, innovation and investment and will lead to business closures and job losses<br>- Will negatively affect/even lead to the end of tobacco and OTP industries (including retailers)	TTCs, OTP importer, OTP retailers, retailer, trade associations (tobacco manufacturers, OTP manufacturers, OTP importers)
		- Will affect the UK's reputation and reduce its standing in the world	OTP retailer
		Negative impacts on business will affect the wider economy and reduce GDP<br>- Loss of tax revenue	TTC, OTP retailer, trade association (tobacco manufacturers), think tank
		- Inclusion of OTPs will have negative impact on tourism	OTP importers, OTP retailers, trade associations (OTP importers)
		In LMICs, policy will impede economic development and make LMICs less competitive<br>- Financial impact on Central American cigar producers	OTP importer
		Policy implementation will increase administrative cost to governments	n/a
		Policy will discourage foreign investment in the country	n/a
		Corporations will not be able to support or invest in social justice projects	n/a
	Policy will fail and have perverse consequences	Policy will not work or has not worked elsewhere<br>- Prohibition does not work or has minimal impact (referencing Bhutan, Islamic State, New Zealand and South Africa)	TTCs, think tank, trade association (OTPs), organisation advocating for harm reduction
		- Policy would push sales online, making it harder to enforce OTP age restrictions	OTP importer
		- Creating an "unenforceable law" could undermine "respect for the law in general"	Think tank
		Policy is a blunt or simplistic instrument and will not achieve nuanced change<br>- Including HTPs in a ban would stop adults who smoke from switching to less harmful products	TTCs, think tank
		- Including cigars/cigarillos in a ban will not protect young people as OTPs are not used by youth/ will not help the to achieve government's objective	OTP retailer, trade associations (OTP manufacturers, OTP importers, OTP)
		Policy will cause confusion and fear<br>- Will create misunderstanding about the relative risks of different tobacco and nicotine products	TTCs
		- Will encourage threatening or violent behaviour towards shop workers	Retailer, trade association (tobacco manufacturers), retail magazine

		Policy will increase illicit trade and smuggling<br>- Will lead to increase in illicit trade of unregulated combustible cigarettes and OTPs	TTCs, OTP manufacture, OTP retailer, retailer, trade associations (cigarette manufacturer, OTP), think tank, smokers' rights group, organisation advocating for harm reduction, retail magazine
		*Policy will criminalise retailers and adults buying tobacco products for other adults**	TTC, OTP retailer, trade association (OTP)
**The acceptable, 'good' solution: individual-focused, corporate supported**	Solutions should target individuals, not whole populations	Solution is to help individuals or 'problem' sub-populations to change their behaviours through information, health education and promotion	
		- Encouragement for adults who smoke to switch to less harmful products is needed, including though education campaigns	TTCs, trade association (tobacco manufacturers), organisation advocating for harm reduction
		Solution is to 'treat' problematic consumption using targeted interventions and 'harm reduction' approaches	
		- Targeted youth prevention education is needed	Retailer, trade associations (tobacco manufacturers, OTP manufacturers, OTP), retail magazine
		- Stricter repercussions for retailers illegally selling tobacco to minors are needed	TTCs, trade associations (cigarette manufacturers, OTP, retailers), think tank
	Solutions should be self-regulatory and not disrupt business	Preferred solutions that cause less disruption to business:	
		- Exemption for HTPs	TTCs, think tanks, smokers' rights group, organisations advocating for harm reduction
		- Exemption for OTPs	OTP manufacturer, OTP importers, OTP retailers, trade association (OTP manufacturers, OTP importers, OTP retailers), think tank
		- Increase age of sale of tobacco products to 21 instead	TTCs, organisation advocating for harm reduction
		- Staged approach to enforcement i.e. warning letters rather than fines for retailers for first three months	Trade association (retailers)
		- Repeal snus ban	TTC, smokers' rights group, organisations advocating for harm reduction
		Self-regulation and voluntary action by corporations (on advertising, marketing, labelling etc.) are more effective and more compatible with business operations	n/a

*This illustration was not part of the original framework but was added inductively during data analysis.
