# Supplementary material for: The UK Tobacco and Vapes Bill (2023/4): framing strategies used by tobacco and nicotine industry actors faced with an endgame policy (a generational sales ban of tobacco products) and nicotine product restrictions
Source: Tob Control. 2025 Mar 23;35(4):e059207. doi: 10.1136/tc-2024-059207 (PMC13479673; doi:10.1136/tc-2024-059207)
Supplement: Supplementary file 5 [file tc-35-4-s005.pdf]

Supplemental File 5: Actors' framing strategies around measures to tackle youth use of nicotine products

Table 1: Framing strategies regarding nicotine product regulation: the problem, the 'bad' solution and the 'good' solution(s) (based on Ulucanlar et al., 2024)

Framing the Policy Space	Frame-supporting claims	Illustrations/Examples	Types of actors using claims
The 'trivial' and 'individual' problem: created by a minority of consumers *and other actors**	*Problem* is not caused by Industry's products/services	Products/ingredients/services are harmless or cause minimal problems	
		- E-cigarettes are a safer alternative to combustible cigarettes	TTC, NP consultancy
		- Nicotine pouches are safer than nicotine products that are inhaled	TTC and smokers' rights group
		- Non-nicotine e-cigarettes are safer than those with nicotine	Smokers' rights group
		Industry's products/ingredients have been misclassified/confused with other, genuinely harmful products/ingredients.	
		- Those selling illegal e-cigarettes and sell e-cigarettes to children are the problem.	NP manufacturer
		Industry's products/services contribute to health, wellbeing and enjoyment of life	
		- E-cigarettes play an important role in quitting	NP manufacturers, organisation advocating for harm reduction
		Health problems have complex causes that cannot be traced to industry products or services alone.	n/a
		Industry's products/services are aligned with cultural norms and practices and are used responsibly by the majority	n/a
	Problem arises from consumption patterns of atypical minorities	Health harms result from individuals' or sub-populations' wrong or uninformed choices and irresponsible behaviours AND Health harms result from cognitive problems or physical/mental health problems.	
		- Youth NP use linked to those more predisposed to risky behaviours ("certain personality traits")	Organisation advocating for harm reduction
		- Most youth NP use about curiosity/ experimentation, not addiction	NP consultancy
	Health harms are exaggerated	Health harms only affect a minority and are exaggerated by the public health community.	n/a
		There are far more serious and urgent health problems that government should prioritise instead	n/a
The unacceptable, 'bad' solution: whole population, statutory	Policies are unnecessary & unacceptable	Existing regulation is sufficient and should be better enforced before new measures are introduced<br>Better enforcement of current age-of-sale rules and penalisation of incompliant sellers	TTCs, NP manufacturers, trade associations (retailers, NP), think tanks, organisations advocating for harm reduction
		Policy is disproportionate to the problem.	
		- Disproportionate to treat NPs like tobacco products given they are safer	NP manufacturer
		- Regulation disproportioned compared to other products (like alcohol)	TTC, NP manufacturer
		Policy is not evidence-based	TTCs, NP manufacturer, think tanks, organisations advocating for harm reduction.

		Policy is unnecessary because corporations are successfully self-regulating and initiating public health interventions.	n/a
		Policy is out of line with global standards and other countries' policies.	n/a
		Policy is regressive and discriminatory.	n/a
	Policies/policy formulation contravene norms, rules & laws	Government has not sufficiently consulted industry or other groups.	Trade association (NP), NP consultancy (see also supplementary file 2)
		Policy is unconstitutional, impedes basic rights (eg, the right to free speech) and curtails basic freedoms of a legal business.	
		Packaging restrictions impede companies' rights to differentiate their products	Think tank
		Flavours restrictions impede right of choice of adult who smoke	TTC
		Policy is illegal (eg, it violates terms of international trade and investment agreements)	
		PP, display ban, flavour ban would violate intellectual property and international trade agreements	TTC
		Government has failed to conduct comprehensive social and economic impact assessment.	n/a
		The body proposing regulation has no legal authority to do so.	n/a
	Policies will lead to losses for businesses, economy & society	Policy will be *difficult or impractical* to implement (cost)effectively.	
		- Standardised packaging of NPs would harm the industry	TTC
		- An NP display ban would be costly for retailers	TTCs, NP manufacturer, smokers' rights group
		Policy will reduce competitiveness, innovation and investment and lead to business closures and job losses	
		- A NP display ban will make specialist shop unviable	TTC
		- A NP display ban will make the nicotine industry dominated by the tobacco industry	Trade association (NP)
		- A display ban will disproportionally affect those with smaller market shares as well as new market entrants	TTC
		- Removal of branding and display ban would discourage producers to improve the product	TTC, smokers' rights group
		Negative impacts on business will affect the wider economy, reducing GDP.	
		- Standardised packaging of NPs will harm the Government and industry	TTC
		Policy implementation will increase administrative cost to governments.	n/a
		Policy will discourage foreign investment in the country.	n/a
		In LMICs, policy will impede economic development and make LMICs less competitive.	n/a
		Corporations will not be able to support or invest in social justice projects.	n/a
	Policy will fail & have perverse consequences	Policy will not work or has not worked elsewhere.	
		- Any NP restrictions will lead to resurgence in smoking	TTCs, trade association (NP), think tanks, smoker's rights group, organisations advocating for harm reduction, NP consultancy
		- Restricting flavours could lead/has led to a resurgence in smoking	NP manufacturers, trade association (NP)
		- Standardised packaging for NPs will reduce switching	NP manufacturer
		Policy is a blunt or simplistic instrument and will not achieve nuanced change.	
		- Restricting packaging will not work as packaging is not the key catalyst for youth use	Trade association (NP), organisation advocating for harm reduction

		<u>Policy will cause confusion or fear</u><br>- Standardised packaging, flavour and/ or display ban will lead to confusion and misconception among adults who smoke and will discourage them from switching	 TTCs, NP manufacturers, trade association (NP), think tank, organisations advocating for harm reduction
		<u>Policy will increase illicit trade and smuggling or encourage cross-border shopping.</u><br>- Policy, especially flavour restrictions and standardised packaging, will lead and/or have led to a considerable increase in illicit trade<br>*<u>Display ban will discourage retailers stocking the products*</u>*	 TTCs, think tanks, organisations advocating for harm reduction<br>Smokers' rights group
The acceptable, 'good' solution(*s)*: individual-focused, corporate supported	Solutions should target individuals, not whole populations	<u>Solution is to help individuals or 'problem' sub-populations to change their consumption behaviours through information, health education and promotion.</u><br>- Adults who smoke should be given accurate information about NPs so they can make an informed choice or are encouraged to switch, and particularly keeping them on display will help<br>- Education material should be provided at the point of sale<br>- There should be government approved messaging on risk profile of vapes compared to cigarettes and benefits of e-cigarette use<br><u>Solution is to 'treat' problematic consumption using targeted interventions and 'harm reduction' approaches</u><br>- Youth should be educated about harm reduction<br>- Communities should be educated about illegal NPs and non-compliant retailers and encouraged to report misconduct.<br>- Non-compliant vendors should be penalised (more severely)<br>*<u>Solution is to help individuals whose problems need to be addressed more holistically*.</u>*<br>- "A more holistic approach, addressing the underlying factors that predispose youth towards risky behaviours [youth use of cigarettes and NPs], is essential"	 TTCs, NP manufacturer. trade association (retailers), think tanks, organisations advocating for harm reduction<br>Organisation advocating for harm reduction<br>TTC<br><br>NP consultancy<br>NP manufacturer<br>TTC and NP manufacturer<br><br>Organisation advocating for harm reduction
	Solutions should be self-regulatory & not disrupt business	<u>Self-regulation and voluntary action by corporations are more effective and more compatible with business operations.</u><br>- Policies, especially, on flavours, should be a matter of market forces and not government<br>- Code of conduct already has clear commitment to supplying products with accurate flavour descriptors<br>- We should develop self-regulatory frameworks for packaging and flavour descriptors based on existing regulations<br>- Regulation of nicotine pouches should build on industry voluntary framework rather than mirroring government regulation on e-cigarettes.	 Smokers' rights group<br>NP manufacturer<br>Organisations advocating for harm reduction<br>TTC

*These illustrations were not part of the original framework but was added inductively during data analysis.
